# Multi-Factor Operating Condition Recognition Using 1D Convolutional Long Short-Term Network

**DOI:** 10.3390/s19245488

**Published:** 2019-12-12

**Authors:** Zhinong Jiang, Yuehua Lai, Jinjie Zhang, Haipeng Zhao, Zhiwei Mao

**Affiliations:** 1 Key Lab of Engine Health Monitoring-Control and Networking of Ministry of Education, Beijing University of Chemical Technology, Beijing 100029, China; jiangzn@mail.buct.edu.cn (Z.J.); lyhlaibit@163.com (Y.L.); zhangjinjie@mail.buct.edu.cn (J.Z.); 18810272291@163.com (H.Z.); 2 Beijing Key Laboratory of High-End Mechanical Equipment Health Monitoring and Self-Recovery, Beijing University of Chemical Technology, Beijing 100029, China

**Keywords:** diesel engine, condition recognition, CNN, LSTM, adaptive dropout

## Abstract

For a diesel engine, operating conditions have extreme importance in fault detection and diagnosis. Limited to various special circumstances, the multi-factor operating conditions of a diesel engine are difficult to measure, and the demand of automatic condition recognition based on vibration signals is urgent. In this paper, multi-factor operating condition recognition using a one-dimensional (1D) convolutional long short-term network (1D-CLSTM) is proposed. Firstly, a deep neural network framework is proposed based on a 1D convolutional neural network (CNN) and long short-Term network (LSTM). According to the characteristics of vibration signals of a diesel engine, batch normalization is introduced to regulate the input of each convolutional layer by fixing the mean value and variance. Subsequently, adaptive dropout is proposed to improve the model sparsity and prevent overfitting in model training. Moreover, the vibration signals measured under 12 operating conditions were used to verify the performance of the trained 1D-CLSTM classifier. Lastly, the vibration signals measured from another kind of diesel engine were applied to verify the generalizability of the proposed approach. Experimental results show that the proposed method is an effective approach for multi-factor operating condition recognition. In addition, the adaptive dropout can achieve better training performance than the constant dropout ratio. Compared with some state-of-the-art methods, the trained 1D-CLSTM classifier can predict new data with higher generalization accuracy.

## 1. Introduction

A diesel engine is a kind of internal combustion engine that converts thermal energy into mechanical energy. It plays an important role in the field of national defense, in the chemical industry, in the marine industry, for nuclear power, and so on. Once a diesel engine fails, it not only causes economic losses directly or indirectly in terms of the shutdown of equipment, but it may also threaten the personal safety of users [1,2]. To enhance the availability of the diesel engine, it is imperative to monitor the engine condition and detect early faults. However, the detection of faults and the diagnosis of diesel engines [3] are not simple tasks due to the complex structure and fickle working conditions. If the operating conditions are not considered in detection and diagnosis activities, it is likely to lead to false alarms or missed detection [4,5]. With the information of operating conditions, the engineering applicability of a fault detection and diagnosis method [6,7,8] can be improved to avoid fatal performance degradation and huge economic losses at an early stage. Unfortunately, most fault detection methods are carried out under stable operating condition to avoiding variable operating conditions. Therefore, condition recognition is an important and urgent task in practical engineering applications.

In a diesel engine, the flywheel is attached to the crankshaft, and they rotate together. They convert the reciprocating motion of the piston into the rotational motion of the crankshaft, which outputs torque for the driving of the car and other power-driven mechanisms. Therefore, the operating conditions of a diesel engine can be determined by two parameters: load and the rotation speed of the crankshaft. The load is the output torque of the engine through the flywheel. However, the multi-factor operating conditions of a diesel engine are difficult to measure in many situations, such as for the power systems of vehicles, propulsion devices of ships, and other dynamic equipment. Therefore, the demand for automatic recognition of multi-factor operating conditions is urgent.

During the operation of a diesel engine, the corresponding status information can be obtained by using vibration analysis [9], oil analysis [10], thermal performance analysis [11], and visual inspection. Vibration is an intrinsic mechanical phenomenon, and the vibration signals contain rich information about the diesel engine’s status; thus, vibration monitoring is a powerful tool for condition recognition, as well as fault detection and diagnosis. In this paper, we aim at recognizing the multi-factor operating conditions of a diesel engine based on vibration signals.

Thanks to the development of computing calculation power and powerful signal processing techniques, the recognition tasks based on vibration signals made great progress. At present, some recognition algorithms based on vibration signals exist, and most of them focus on designing various handcrafted features, fusing multiple features and training different classifiers. In Reference [12], the Hilbert spectrum entropy, which combines the Hilbert spectrum and information entropy, was proposed for the pattern recognition of diesel engine working conditions. In Reference [13], the frequency domain features of vibration signals were extracted for back propagation (BP) and radial basis function (RBF) neural network training to recognize the cylinder pressure. In Reference [14], based on the cylinder head vibration signals measured under stable operating conditions, an engine cylinder pressure identification method using a genetic algorithm with BP neural network was proposed. In Reference [15], combustion evaluation parameters were extracted using time–frequency coherence analysis and the cylinder pressure could be estimated based on the parameters and an RBF neural network. In Reference [16], the measured signal was converted into a crank angle degree signal using the rotational speed monitored by magnetic pickup sensors. Then, a real-time engine load classification algorithm was proposed based on an artificial neural network. 

Most pattern recognition studies focused mainly on single-factor conditions or recognition under stable operating conditions. For single-factor conditions, the number of categories is generally no greater than five. In practical engineering applications, a single factor cannot describe complex operating conditions, and this drawback results in ambiguous boundaries among different operating conditions. As for multi-factor operating conditions, as the number of operating conditions increases, so does the complexity of condition recognition. Simultaneously, as the vibration signals are random, transient, and cyclostationary, and as the corresponding feature extraction requires rich domain knowledge, it is difficult to extract sensitive characteristics of significant importance for multi-factor operating condition recognition.

Over the last few years, with the development of deep learning, many researchers exploited deep neural networks (DNNs) as the feature extractor and classifier [17,18]. Benefiting from the powerful feature extraction ability of neural network, especially convolution neural networks (CNNs) [19], these approaches and their variations exhibit good performance in the related tasks. In Reference [20], time domain and frequency domain feature representations were selected to form a vector to act as the input parameters of a CNN. The trained CNN classifier could diagnose the fault patterns of a gearbox with outstanding performance. In Reference [21], the vibration signals of rolling bearings were analyzed using continuous wavelet transform to get time–frequency representations in grayscale. Then, all compressed time–frequency representations were taken as the input for CNN training, and the trained CNN classifier could identify the faults of rolling bearings with strong generalization ability. In Reference [22], a deep convolutional neural network of up to 38 layers, which could provide high classification accuracy, was proposed for gas classification. For CNN applications with vibration signals, there are different approaches to network input. In other words, the CNN is taken as a classifier, and the input of the CNN is mainly based on other feature extraction methods. At the same time, state-of-the-art CNN models have several parameters, which leads to problems related to storage, computation, and energy cost. In addition, recurrent neural networks (RNNs) and long short-term networks (LSTMs) [23,24] were validated in terms of their performance on one-dimensional (1D) signals. In Reference [25], a CNN and a fully connected neural network were both incorporated into a deep neural network framework to improve LSTM. The framework outperformed the original LSTM for the early diagnosis and prediction of sepsis shock. In Reference [26], an end-to-end model combining a CNN and RNN was proposed for the automatic detection of atrial fibrillation. Compared to the state-of-the-art models evaluated on standard benchmark electrocardiogram datasets, the proposed model produced better performance in detecting atrial fibrillation. The ideas in References [25,26] are very good references for multi-factor operating condition recognition based on vibration signals.

Therefore, a multi-factor operating condition recognition algorithm is proposed herein based on a 1D CNN and LSTM. In the proposed neural network framework, the 1D CNN was designed to extract local features of vibration signals through 1D convolution, and the LSTM was designed to describe the temporal relationship between local features. The contributions of this paper are summarized as follows:

1. A multi-factor operating condition recognition method is proposed using a 1D convolutional long short-term network (1D-CLSTM). As far as we know, this is the first study to combine a 1D CNN and LSTM to recognize operating conditions based on a time series of vibration signals;
2. Considering the particularity of engine vibration signals, batch normalization (BN) is introduced to regulate the input of some layers by fixing the mean value and variance of input signals in each convolutional layer;
3. Adaptive dropout is proposed for improving the model sparsity and preventing overfitting;
4. The designed 1D convolutional long short-term network (1D-CLSTM) classifier can achieve high generalization accuracy for recognizing multi-factor operating conditions.

The rest of this paper is organized as follows: Section 2 presents the test bench of a diesel engine and the experimental data acquisition. Section 3 introduces the technical background for the 1D CNN and LSTM. Section 4 describes the designed 1D-CLSTM and the flowchart of the multi-factor operating condition recognition algorithm. Section 5 shows the training performance of the designed 1D-CLSTM classifier, with generalizability verification, a performance comparison with different methods, and a training performance comparison with different dropout ratios. Finally, conclusions and future prospects are presented in Section 6.

## 2. Experiment and Vibration Signal

### 2.1. Test Bench of Diesel Engine

For data acquisition, a four-stroke diesel engine numbered TBD234 (produced by Henan Diesel Engine Industry Co. Ltd., Luoyang, China) was used and tested in different operating conditions. The parameters of the diesel engine are shown in Table 1.

**Figure 1:**
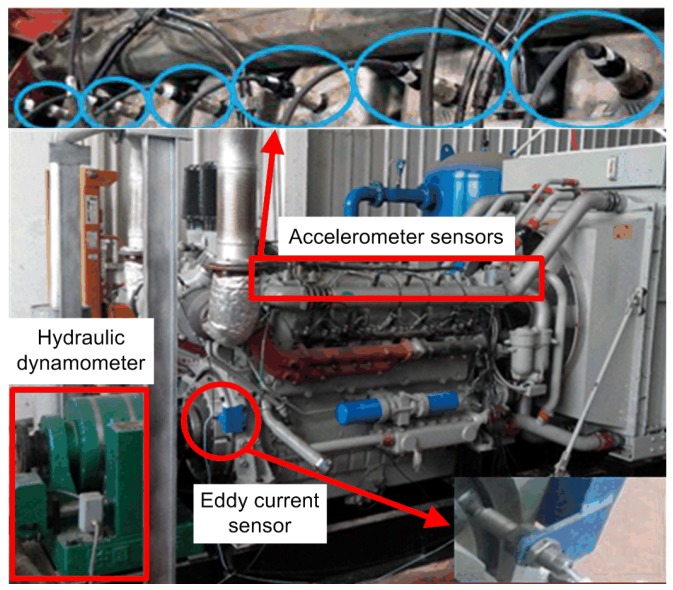
Test bench of the diesel engine.

As shown in Figure 1, 12 acceleration sensors were arranged on the surface of corresponding cylinder heads to monitor the status information of the diesel engine in the running state. The vibration signals formed the basis for the multi-factor operating condition recognition of the diesel engine. Moreover, an eddy current sensor was arranged on the flywheel to collect the information of rotating speed. In addition, a hydraulic dynamometer was connected with the output end of the diesel engine to adjust the load.

**Figure 2:**
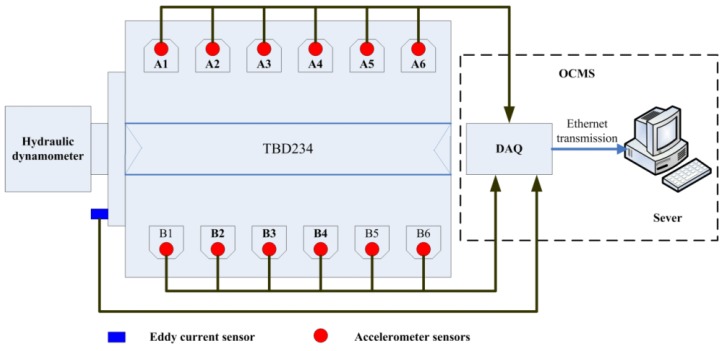
Structure diagram of the online condition monitoring system (OCMS) of the diesel engine.

All signals were measured using an online condition monitoring system (OCMS) at a sampling frequency of 51.2 kHz per channel in all tests, and the results were saved to a server through Ethernet transmission. The structure diagram of the OCMS of the diesel engine is shown in Figure 2.

### 2.2. Experimental Data Acquisition

To extract vibration data under different operating conditions, the engine was run at different levels of operating conditions. The representative operating conditions are listed in Table 2. 

**Figure 3:**
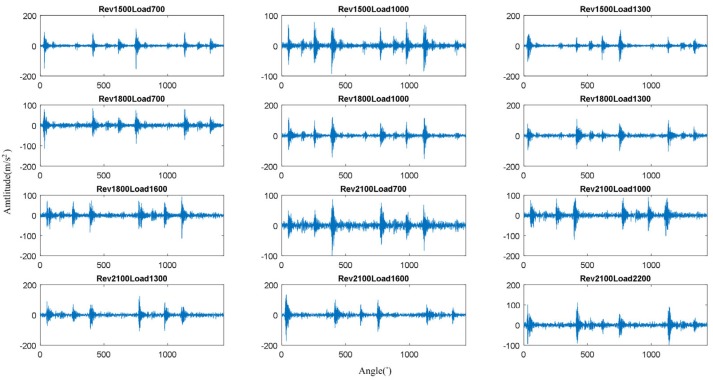
Vibration signals of 12 different operating conditions.

Through the OCMS, vibration signals of different operating conditions could be measured. The vibration signals of 12 different operating conditions are shown in Figure 3.

The signals in Figure 3 represent two complete periodic vibration signals, with a certain cyclic fluctuation in the angular domain. When fire combustion and closing of the intake valve and exhaust valve occur, an obvious excitation response is produced in the corresponding phase. Due to the different ignition phase points of different cylinders, the corresponding combustion excitation occurs at different positions. As the amplitude of the vibration signal features large randomness, the vibration signal of a diesel engine can be considered a non-periodic and non-stationary signal. This characteristic of the vibration signal greatly increases the difficulty of multi-factor operating condition recognition.

## 3. Technical Background

In this study, a deep neural network framework is proposed based on a 1D CNN and LSTM for multi-factor operating condition recognition. For the vibration signal in the form of a time series, a 1D CNN was adopted to extract local features of vibration signals through a 1D convolution kernel. Then, an LSTM was adopted to describe the temporal relationship between local features through a memory unit and gate mechanism. In this way, the combination of the 1D CNN and LSTM could perform well for the analysis of vibration signals.

### 3.1. 1D CNN

A typical CNN [19] contains three types of network layers: a convolutional layer, pooling layer, and fully connected layer. Some excellent variants of CNN were proposed, such as LeNet-5 [27], AlexNet [19], and VGG-16 [28]. The image recognition ability of these CNN variants is outstanding, and they achieved remarkable results. In CNNs, the receptive field, weight sharing, and pooling can greatly reduce the complexity of the network.

**Figure 4:**
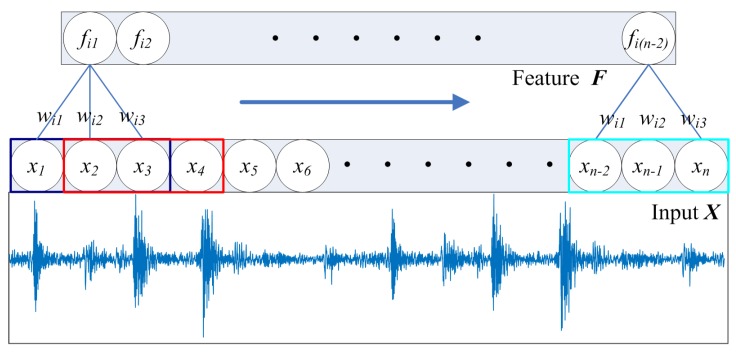
Temporal convolution.

It was proven that a 1D CNN can be applied to the time series analysis of sensor data. In 1D CNNs, features can be extracted from segments through 1D convolution, which is a weighted sum operation between the weight matrix and the vibration data in each segment, with the addition of the overall bias. Every convolution extracts a feature from a local receptive field, and the window of the convolution kernel slides across the entire input sequence with a fixed step to achieve all features. The weight sharing exists to maintain the weights of the convolution kernel in the sliding process. As shown in Figure 4, the size of the *i*-th convolution kernel is shown, featuring weights (*w_i_*_1_, *w_i_*_2_, *w_i_*_3_) in a 1 × 3 format, with the bias left out for clarity. The corresponding feature vector ***F*** (*f_i_*_1_, *f_i_*_2_, *f_i_*_3_, …, *f_i_*_(_*_n_*_−2__)_) can be obtained from the input signal ***X*** (*x*_1_, *x*_2_, *x*_3_, …, *x*_n_) with one step of the convolution kernel. 

Mathematically, this can be expressed as shown in Equation (1).

(1) $$f_{ij}=\Phi \left(b_{i}+\sum_{k=1}^{m}w_{ik}\cdot x_{j+k-1}\right),$$

where *m* is the size of the convolution kernel, *f_ij_* is the output of the *j*-th neuron of the *i*-th filter in the hidden layer, Φ is the activation function, and *b_i_* is the overall bias of the *i*-th filter.

Convolution kernels of different sizes can extract features of different granularity [29]. Usually, the first convolutional layer may only extract some low-level features, and more complex features can be extracted from low-level features by stacking network layers.

As the pooling operation can maintain the variance of the translation, rotation, and scale, the pooling layer is set following each convolutional layer to retain the main features. Meanwhile, it can reduce the number of parameters to prevent overfitting and improve the generalizability of the model. In a pooling layer, the features obtained from the activation function are cut into several regions, and the maximum/average values can be taken as the new features to realize dimension reduction. By repeating operations as described above, features can be extracted continuously to improve the generalizability of the CNN. 

Enough sensitive important features can be extracted by alternating convolutional and pooling layers, and the fully connected layers can map the distributed feature representation to the sample markup space. Finally, the output layer with a softmax activation function is used for classification.

### 3.2. LSTM

**Figure 5:**
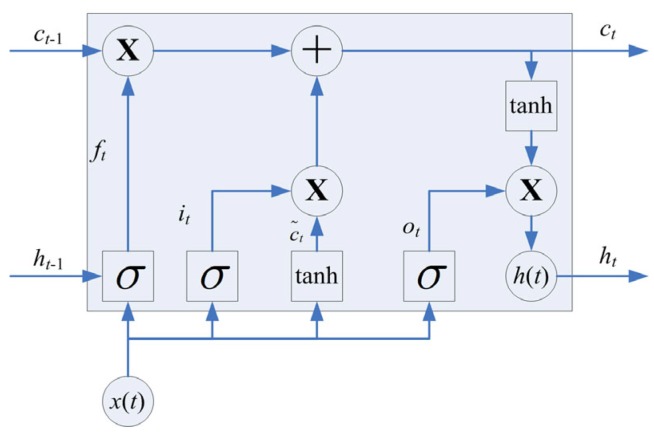
Memory cell of a long short-term memory network (LSTM).

A recurrent neural network (RNN) is a kind of neural network which can be used for sequential data analysis, while the LSTM is a specific kind of RNN. Compared with a traditional RNN, a memory cell and gating mechanism are introduced to deal with the existence of gradient disappearance and gradient explosion during the training of long sequences. The gating mechanism can be used to control the transfer state, which is designed to remember the important information and forget the unimportant information. The memory cell of an LSTM is shown in Figure 5.

As shown in Figure 5, the memory cell of an LSTM is made up of an input gate, output gate, and forget gate. The sigmoid activation function is used in the forget gate to control the weight of information that can be passed, whereas the tanh activation function is used in the input gate to deal with the input at the current sequence position, and the sigmoid activation function is used in the output gate to update the output based on the results of the input gate and forget gate. Mathematically, the parameters of the LSTM can be updated as shown in Equation (2).

(2) $$\begin{array}{l} i_{t}=\sigma \left(W_{xi}x_{t}+W_{hi}h_{t-1}+b_{i}\right);\\ f_{t}=\sigma \left(W_{xf}x_{t}+W_{hf}h_{t-1}+b_{f}\right);\\ o_{t}=\sigma \left(W_{xo}x_{t}+W_{ho}h_{t-1}+b_{o}\right);\\ {\tilde{c}}_{t}=\tanh\left(W_{xc}x_{t}+W_{hc}h_{t-1}+b_{c}\right);\\ c_{t}=f_{t}\cdot c_{t-1}+i_{t}\cdot {\tilde{c}}_{t};\\ h_{t}=o_{t}\cdot \tanh\left(c_{t}\right). \end{array}$$

where *x_t_* is the input of a sequence, *c_t_*_−1_ is the last state, and *h_t_*_−1_ is the output of the last memory cell. The state *c_t_* and output *h_t_* of the current memory cell can be obtained after parameter update calculation.

## 4. Methodologies

In this section, the 1D-CLSTM is firstly constructed for multi-factor operating condition recognition, and then adaptive dropout is proposed. Moreover, the flowchart of the multi-factor operating condition recognition method is introduced.

### 4.1. 1D Convolutional Long Short-Term Network

#### 4.1.1. Overall Architecture

**Figure 6:**
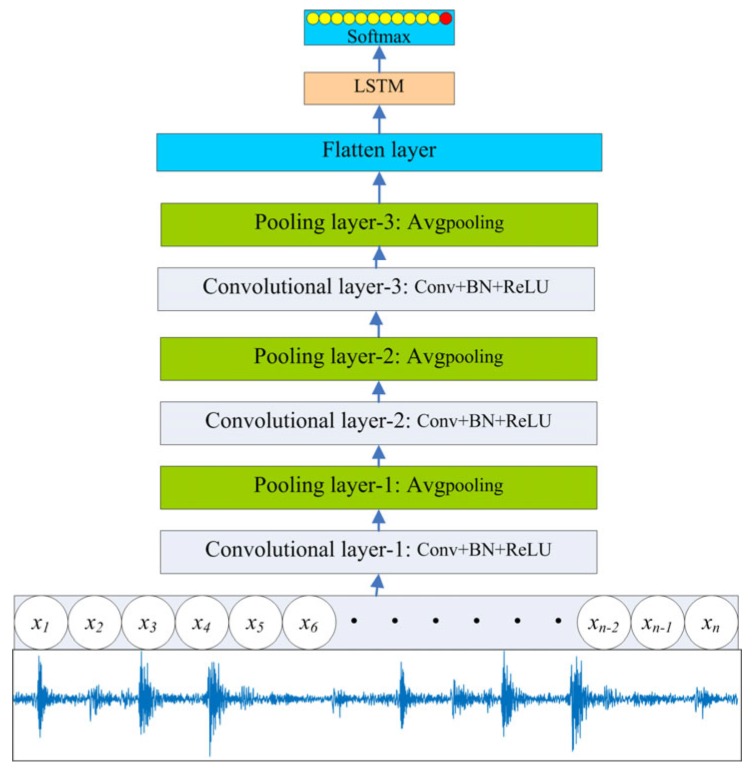
Overall architecture of the one-dimensional convolutional long short-term network (1D-CLSTM).

As described above, the features extracted by different neural networks have different characteristics. The 1D CNN can obtain the features of a receptive field through convolution, but the temporal relationship of the vibration signal is ignored as a result of the size of the convolution kernel. As for the LSTM, a temporal relationship can be described through the memory cell and gating mechanism. Therefore, the multi-factor operating condition recognition algorithm 1D-CLSTM is proposed based on a 1D CNN and LSTM. In the proposed neural network framework, the 1D CNN was designed to extract local features of vibration signals through 1D convolution, and the LSTM was designed to describe the temporal relationship between local features. The overall architecture of the 1D-CLSTM is shown in Figure 6.

#### 4.1.2. Architecture Design

According to the sampling frequency of the monitoring system and different operating conditions introduced in Section 2, a signal segment with a length of 4096 can be determined to contain all the information in a cycle. The crankshaft of a four-stroke diesel engine rotates 720 degrees to complete a cycle, which means complete energy conversion. Therefore, the minimum receptive field can be defined as a degree in the angular domain. Moreover, the size of the CNN filter in the first convolutional layer can be set to an odd number greater than 4096/720.

Considering the particularity of a vibration signal, which is a non-periodic and non-stationary signal, BN [30] is vital for regulating the input of some layers by fixing the mean value and variance of input signals of each convolutional layer, through which the features can maintain the same distribution in the training process of the 1D-CLSTM. Upon increasing the number of layers in a neural network, the decreasing convergence rate often leads to gradient explosion or gradient disappearance, and BN is an excellent solution. Therefore, the convolution is followed by BN in each convolutional layer. In all convolutional layers, the rectified linear unit (ReLU) activation function is adopted, and BN occurs in front of the ReLU activation function. In other words, the results of BN are the input of the ReLU activation function. The ReLU activation function makes the output of some neurons equal to 0, which results in sparsity of the network, thereby reducing the interdependence of parameters and alleviating the occurrence of the overfitting problem. The average values of features obtained from the ReLU activation function are taken as the new features to realize dimension reduction in a pooling layer. The designed 1D-CLSTM begins with a sequence input, after which the features can be extracted by alternate convolutional layers and pooling layers.

A complete periodic signal contains different sequential excitation responses; thus, the sequence length processed by the LSTM can be determined according to the degree of excitation responses in the angular domain. When the degree of an excitation response in the angular domain is 15, the number of LSTM units can be chosen to be greater than 720/15. Following the final pooling layer, there is a flattening layer to reshape the tensor as the input of the LSTM with 73 units. In order to accelerate the convergence process of 1D-CLSTM training, adaptive dropout is applied. Finally, the output layer with a softmax activation function is used for multi-class classification. The structural parameters of the 1D-CLSTM are shown in Table 3.

#### 4.1.3. Adaptive Dropout

Dropout is widely used for improving model sparsity and preventing overfitting in model training. The learning process of the 1D-CLSTM for multi-factor operating condition recognition is an iterative one. On account of the mutual influence among interconnected neurons, every iteration is a greedy search, whereby we find the best connections. That is, a connection may be unimportant due to the existence of some others, but it becomes important once the others are removed. Therefore, the adaptive dropout ratio is proposed to deal with this problem.

The most popular Bernoulli dropout technique [31] can be applied to neurons or weights. Assuming the input of a weight or neuron as ***X***, the output as ***Y***, the dropout probability as P(α), and the weight matrix as ***W,*** each neuron is probabilistically dropped at each training step, as defined in Equation (3).

(3) Y=(X·P)W.

Each weight in the weight matrices is probabilistically dropped at each training step, as defined in Equation (4).

(4) Y=X(W·P).

Usually, the dropout ratio α is constant for generating random network structures (for example, 0.5). However, the model capacity is constantly changing within the 1D-CLSTM training. Therefore, the dropout ratio needs to be adaptive to the current network. Neurons or weights are dropped temporarily during training and dropped forever after pruning to solidify the network structure. Compared with the original network structure, the parameters of the current network become sparse after pruning, and the dropout ratio should be reduced.

Assuming that the connection between the input layer and output layer is fully connected, the number of connections can be calculated as shown in Equation (5).

(5) $$C_{i}=N_{i}N_{o}.$$

Since dropout works on neurons, taking *C_io_* as the original network and *C_ic_* as the current network, the dropout ratio α can be adjusted according to Equation (6).

(6) $$\alpha_{c}=\frac{\alpha_{o}N_{o}}{\left(N_{o}+1\right)}\sqrt{\frac{C_{ic}}{C_{io}}},$$

where $\alpha_{c}$ represents the dropout rate of the current network, and $\alpha_{o}$ represents the dropout rate of the original network.

#### 4.1.4. Implementation

The loss function, which measures the degree of difference between the predicted value and actual value, is a non-negative real value function. A smaller loss function denotes better robustness of the model. Cross-entropy is frequently used for loss calculation in neural network training, as shown in Equation (7).

(7) $$\mathrm{loss}=-\sum_{i=1}^{n}y_{i}\log\left(y_{i\_}\right),$$

where *y_i_* represents the predicted value, *y_i__* represents the actual output, and *n* is the number of training samples.

In the training of the 1D-CLSTM designed for multi-factor operating condition recognition, the learning rate was set to 0.001. Through iterative calculation, the loss of 1D-CLSTM decreased continuously and eventually became stable. Then, the weight of 1D-CLSTM was fixed, allowing the 1D-CLSTM classifier to be used for multi-factor operating condition recognition.

To make the training of the 1D-CLSTM model more efficient and achieve better performance, the training techniques described below were introduced.

Mini-batch gradient descent. Considering the huge calculation in network training, a batch sample was adopted in the training process, and the batch size was set to 128. The batch sample strategy uses less memory and achieves a faster training speed than full batch learning. Compared with stochastic gradient descent, mini-batch gradient descent is more efficient. Compared with batch gradient descent, mini-batch gradient descent can achieve robust convergence to avoid local optimization. Therefore, mini-batch gradient descent was taken as the optimizer to minimize the loss and adjust the weights in the designed 1D-CLSTM.

Early termination. In the process of model training with the training set, the performance of the model is also evaluated with the validation set. The validation error decreases in the beginning as the training error decreases. After a certain number of training steps, the training error still decreases, but the validation error no longer decreases. Therefore, early termination can act as a regulator and effectively avoid overfitting of the model. Once the validation error stops decreasing, the early termination of model training can be enforced in the training of the 1D-CLSTM.

### 4.2. Multi-Factor Operating Condition Recognition

**Figure 7:**
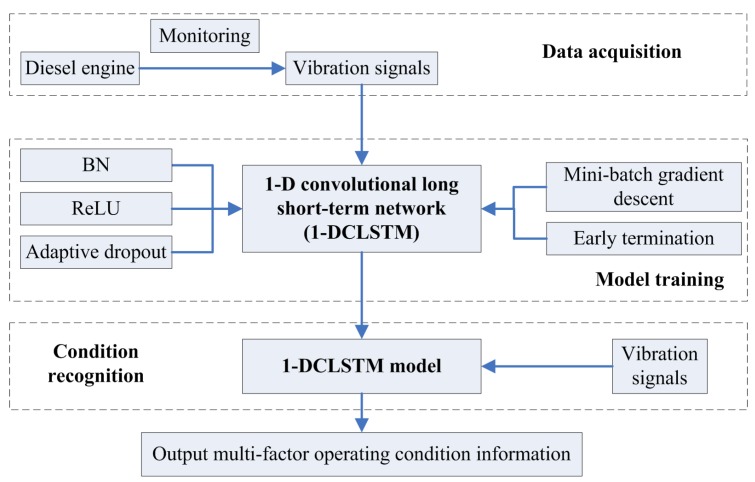
The flowchart of the condition recognition method.

To determine the multi-factor operating condition information of a diesel engine, a condition recognition method using 1D-CLSTM is proposed. Firstly, acceleration sensors were used to monitor the status information of a diesel engine under different operating conditions. Considering the characteristics of the vibration signal, some performance improvement techniques were adopted in the 1D-CLSTM, such as BN, ReLU activation function, adaptive dropout. Moreover, mini-batch gradient descent and early termination were adopted in the training of 1D-CLSTM to achieve a fast training speed and avoid overfitting of the model. Accordingly, the 1D-CLSTM could be trained using supervised learning. After training, the trained 1D-CLSTM classifier could be used for the classification of multi-factor operating conditions. The flowchart of the multi-factor operating condition recognition method is shown in Figure 7.

## 5. Experiments

According to the flowchart shown in Figure 7, the training performance of the designed 1D-CLSTM is presented below. After training, the performance of 1D-CLSTM using vibration signals for multi-factor operating condition recognition was evaluated. Moreover, the vibration signals measured from another kind of diesel engine were applied to verify the generalizability of the proposed approach. Finally, the results of the proposed approach for multi-factor operating condition recognition were compared to other classification algorithms to verify that the designed 1D-CLSTM with strong generalizability could provide higher classification accuracy. The 1D-CLSTM model was written using Python 3.6 with TensorFlow and run on Window 10 with an NVIDIA Quadro P6000.

### 5.1. Training Performance of the Designed 1D-CLSTM

**Figure 8:**
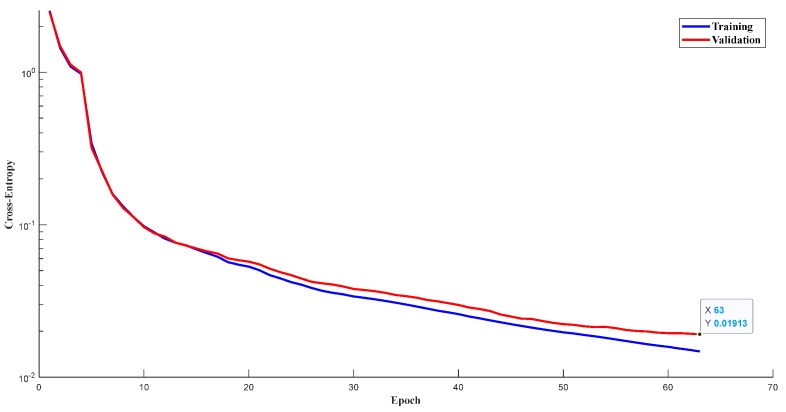
Losses of training set and validation set.

**Figure 9:**
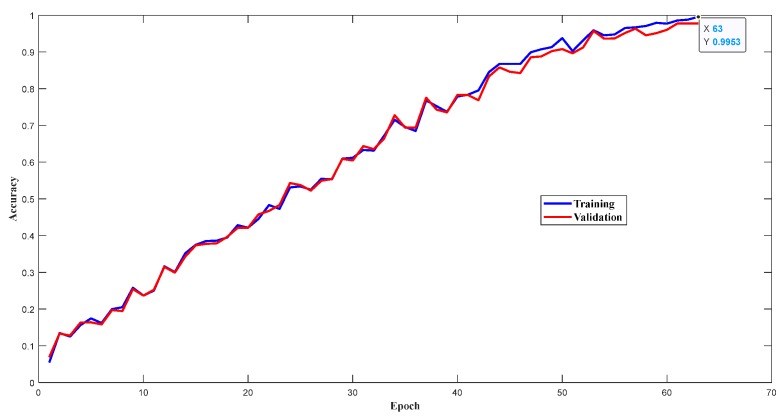
Accuracies of training set and validation set.

The vibration signals were in the form of a time series, used as the input data for training the designed network, with a total of 7200 samples. The whole dataset was randomly divided into two sets: 80% for training and 20% for validation. In other words, the training set had 5760 samples, and the validation set had 1440 samples. With the continuous iterative training of 1D-CLSTM, the losses of the training set and validation set decreased as the number of epochs increased, as depicted in Figure 8. On the contrary, the accuracies of the training set and validation set continuously improved, as depicted in Figure 9. According to the early termination, the model training stopped when the loss of the validation set stopped decreasing. The training of 1D-CLSTM stopped at the 63rd epoch when the cross-entropy of the validation set was 0.01913 and the accuracy of the training set was 0.9953. Therefore, the corresponding 1D-CLSTM classifier is a desired classification model for multi-factor operating condition recognition.

**Figure 10:**
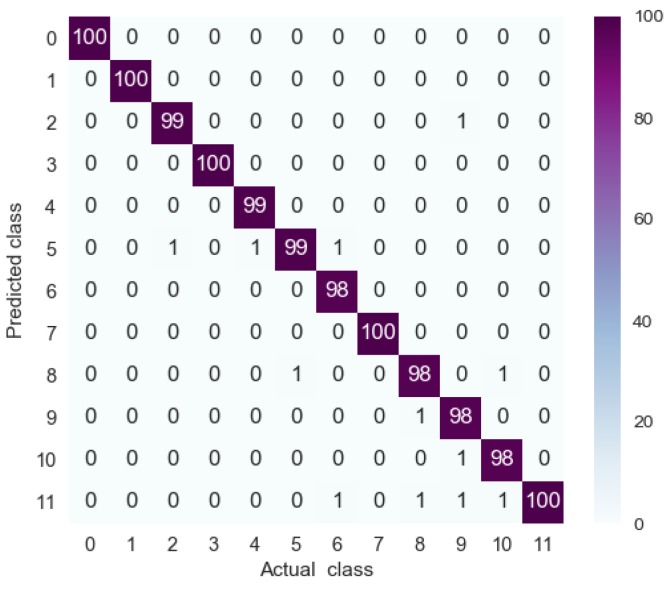
Confusion matrix for testing set.

A confusion matrix, which contains information about actual and predicted classes, was used to describe the generalizability of the 1D-CLSTM classifier [32]. The testing set had a total of 1200 samples, with 100 samples for each operating condition. The confusion matrix for the testing set is shown in Figure 10.

The elements in row *i* and column *j* of the confusion matrix represent the number of times the *j* class was identified as the *i* class. Therefore, only the diagonal elements denote correct recognition. It can be seen from Figure 10 that only 11 samples out of 1200 were misclassified. Therefore, the designed 1D-CLSTM can classify multi-factor operating conditions with an accuracy of 99.08%.

### 5.2. Comparison of Training Performance with Different Dropout Ratios

**Figure 11:**
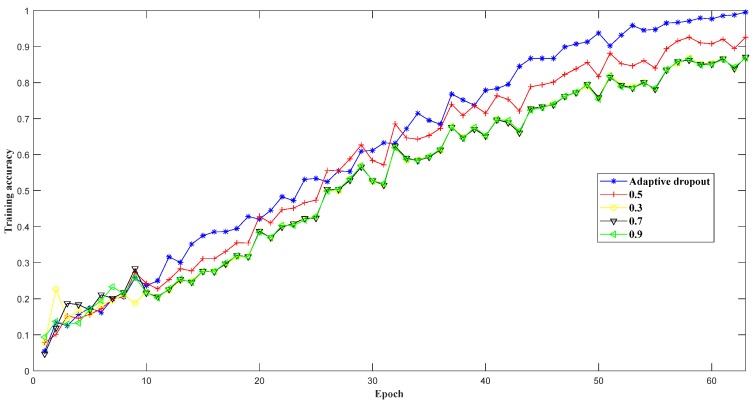
Training accuracy curves with different dropout ratios.

The convergence process in model training is an important factor for achieving a classifier with excellent performance. Dropout serves as an effective approach to improve the model sparsity and prevent overfitting in model training. To find the best connections in the designed 1D-CLSTM, a suitable dropout ratio was very important. Adaptive dropout, due to its flexibility depending on network capacity, is able to maintain the balance between model performance and model sparsity. To check the effect of adaptive dropout, training accuracy curves of different dropout ratios were plotted, as shown in Figure 11. According to the early termination, the model training using adaptive dropout stopped at the 63rd epoch, and the comparison of training performance with different dropout ratios was conducted within 63 epochs.

It can be seen from Figure 11 that the training performance using adaptive dropout was best; thus, adaptive dropout can improve the training performance to achieve the desired model. 

### 5.3. Comparison Analysis

To validate the performance of the designed 1D-CLSTM, the proposed method was compared with the following baseline methods:

1. The k-nearest neighbor (kNN) algorithm, which works with a multi-domain feature set [33]. Based on the multi-domain feature set, the kNN algorithm is more suitable than other statistical learning methods.
2. The support vector machine (SVM), which works with a multi-domain feature set. SVM is a kind of generalized linear classifier that can be used for supervised learning.
3. The 1D LeNet-5, which is a convolutional network that has the same network layers as LeNet-5, i.e., two convolutional layers and two fully connected layers. The corresponding structural parameters are listed in Table 4.
4. The 1D AlexNet, which is a convolutional network that has the same network layers as AlexNet, i.e., five convolutional layers and three fully connected layers. The corresponding structural parameters are also listed in Table 4.
5. The 1D VGG-16, which is a convolutional network that has the same network layers as VGG-16, with 1D convolution kernels adopted. The corresponding structural parameters are also listed in Table 4.
6. A traditional LSTM, which has two layers and 32 LSTM units in each layer.

In Table 4, s represents the stride, and the convolution is followed by BN in each convolutional layer.

For multi-factor operating condition recognition, the class domains of operating conditions are likely to overlap with each other. Our goal was to develop a multi-factor operating condition recognition method that can achieve high generalization accuracy. Therefore, the same vibration data were used for the training and testing with the above methods, and the corresponding model performance is shown in Table 5.

It can be seen from Table 5 that the generalization accuracy of the proposed method was the best. This shows that the 1D-CLSTM learns to predict new data with higher accuracy than other machine learning models and avoids overfitting. In addition, the trained 1D-CLSTM classifier can be used as a good initializer for similar tasks of transfer learning (https://github.com/Larrylyh/Condition_Recognition).

### 5.4. Generalizability Verification

**Figure 12:**
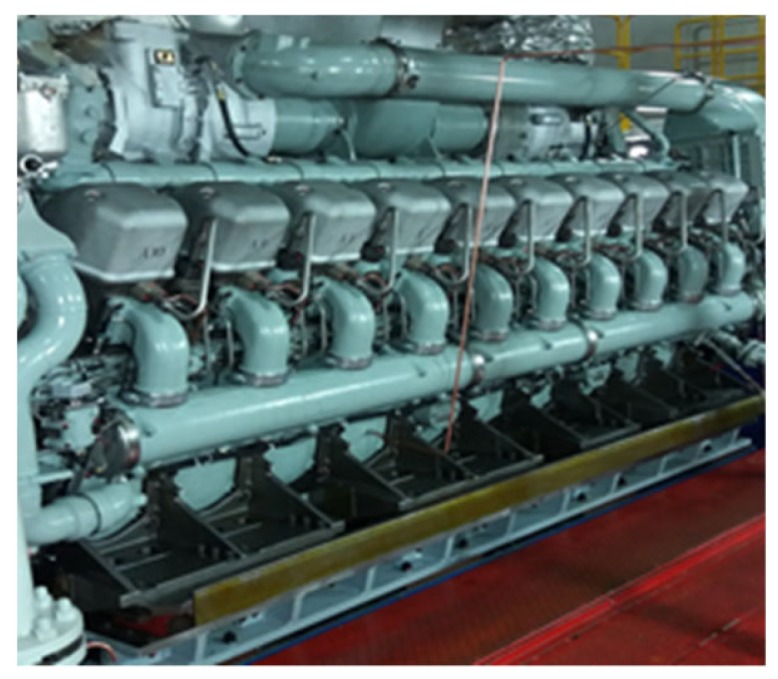
The diesel engine with 20 cylinders.

To verify the generalizability of the proposed approach, the designed 1D-CLSTM was applied to a diesel engine with 20 cylinders (V20DE), which is shown in Figure 12. 

The vibration data under different operating conditions, which are listed in Table 6, were measured.

**Figure 13:**
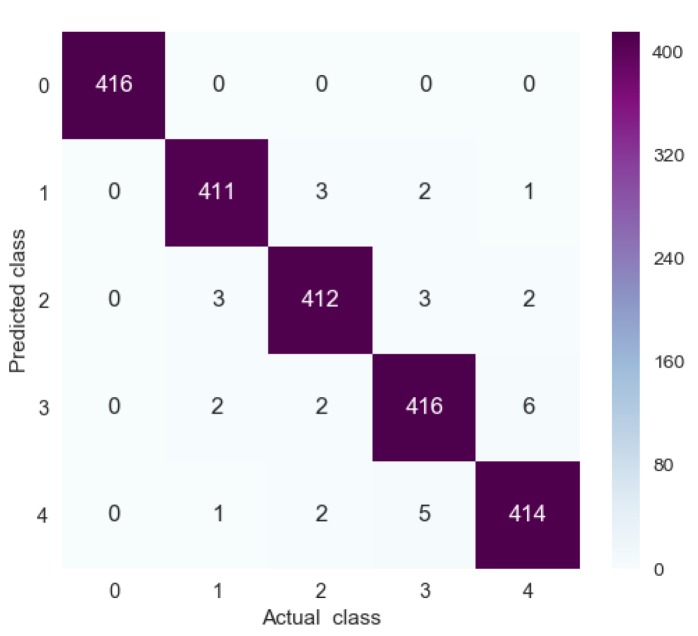
Confusion matrix.

Generally, the data measured from different engine types vary greatly, and the 1D-CLSTM classifier would need to be trained before use. The test set of V20DE contained 2101 samples, and the corresponding confusion matrix is illustrated in Figure 13. As depicted in Figure 13, 32 samples out of 2101 were misclassified, and the corresponding accuracy was 98.48%.

## 6. Conclusions

In this study, an effective approach was proposed for multi-factor operating condition recognition using a 1D convolutional long short-term network. The proposed method was capable of monitoring and automatically recognizing multi-factor operating conditions based on the vibration signal measured on engine cylinder heads. Moreover, the measured vibration signals no longer needed a complex feature extraction process for condition recognition. Subsequently, adaptive dropout was proposed for improving the model sparsity and preventing overfitting in model training. The experimental results proved that the designed 1D-CLSTM classifier is indeed ideal for multi-factor operating condition recognition with high generalization accuracy. At the same time, adaptive dropout could achieve better training performance than a constant dropout ratio. In addition, this method has the potential for application in real-time scenarios because the implementation of the 1D-CLSTM classifier is simple. Last but not least, the trained 1D-CLSTM classifier can be used as a good initializer for similar tasks of transfer learning. In the future, new studies will be conducted on the transition period between the defined operating conditions to obtain a model that can identify continuous operating conditions. Moreover, continuous operating condition recognition can be the basis of fault detection or diagnosis under variable operating conditions.

## Figures and Tables

**Table 1:** Parameters of TBD234 diesel engine.

Item	Parameter
Number of cylinders	12
Shape	V-shaped 60°
Firing sequence	B1-A1-B5-A5-B3-A3-B6-A6-B2-A2-B4-A4
Rating speed	2100 rev/min
Rating power	485 kW

**Table 2:** Operating conditions of the diesel engine.

No.	Rev (rpm)	Load (N·m)	No.	Rev (rpm)	Load (N·m)
1	1500	700	7	1800	1600
2	1500	1000	8	2100	700
3	1500	1300	9	2100	1000
4	1800	700	10	2100	1300
5	1800	1000	11	2100	1600
6	1800	1300	12	2100	2200

**Table 3:** Structural parameters of the one-dimensional convolutional long short-term network (1D-CLSTM).

No.	Network Layer	Size of Convolution Kernel	Stride	Output Dimension
1	Input layer	-	-	4096 × 1
2	Convolutional layer-1	11	1	4096 × 32
3	Pooling layer-1	3	2	2047 × 32
4	Convolutional layer-2	13	1	2047 × 64
5	Pooling layer-2	3	2	1023 × 64
6	Convolutional layer-3	15	1	1023 × 128
7	Pooling layer-3	3	2	511 × 128
8	Flatten layer	-	-	73 × 896
9	LSTM (two layers)	-	-	73
10	Softmax	-	-	12

**Table 4:** Structural parameters of the 1D-CLSTM.

1D LeNet-5	1D AlexNet	1D VGG-16	
Conv1 [1,11] × 64, s = 1	Conv1 [1,11] × 32, s = 1	Conv1 [1,3] × 16, s = 1	Conv9 [1,3] × 128, s = 1
AveragePooling1 [1,3], s = 2	MaxPooling1 [1,3], s = 2	Conv2 [1,3] × 16, s = 1	Conv10 [1,3] × 128, s = 1
Conv2 [1,13] × 128, s = 1	Conv2 [1,5] × 64, s = 1	MaxPooling1 [1,2], s = 2	MaxPooling4 [1,2], s = 2
AveragePooling2 [1,3], s = 2	MaxPooling2 [1,3], s = 2	Conv3 [1,3] × 32, s = 1	Conv11 [1,3] × 256, s = 1
FC1 (1024)	Conv3 [1,3] × 128, s = 1	Conv4 [1,3] × 32, s = 1	Conv12 [1,3] × 256, s = 1
FC2 (512)	Conv4 [1,3] × 128, s = 1	MaxPooling2 [1,2], s = 2	Conv13 [1,3] × 256, s = 1
softmax	Conv5 [1,3] × 128, s = 1	Conv5 [1,3] × 64, s = 1	MaxPooling5 [1,2], s = 2
-	MaxPooling3 [1,3], s = 2	Conv6 [1,3] × 64, s = 1	FC1 (1024)
-	FC1 (1024)	Conv7 [1,3] × 64, s = 1	FC2 (512)
-	FC2 (512)	MaxPooling3 [1,2], s = 2	softmax
-	softmax	Conv8 [1,3] × 128, s = 1	-

**Table 5:** Performance comparison. SVM—support vector machine.

Learning Model	Generalization Accuracy (%)
1D-CLSTM	99.08
LSTM	74.12
kNN with a multi-domain feature set	92.18
SVM with a multi-domain feature set	94.91
1D LeNet-5	94.43
1D AlexNet	97.54
1D VGG-16	98.01

**Table 6:** Operating conditions of V20DE.

No.	Rev (rpm)	Load (kN·m)
1	600	0
2	1100	17.7
3	1500	22.6
4	1500	26.6
5	1500	28.3
